# Rheological and Morphological Properties of Non-Covalently Functionalized Graphene-Based Structural Epoxy Resins with Intrinsic Electrical Conductivity and Thermal Stability

**DOI:** 10.3390/nano10071310

**Published:** 2020-07-03

**Authors:** Maria Rossella Nobile, Marialuigia Raimondo, Carlo Naddeo, Liberata Guadagno

**Affiliations:** Department of Industrial Engineering, University of Salerno, Via Giovanni Paolo II, 132, 84084 Fisciano (SA), Italy; mrnobile@unisa.it (M.R.N.); cnaddeo@unisa.it (C.N.); lguadagno@unisa.it (L.G.)

**Keywords:** epoxy resins, rheology, tunneling atomic force microscopy (TUNA), graphene-based nanocomposites, non-covalent functionalization, morphological analysis

## Abstract

In this paper, a non-covalent π–π interaction between graphene nanoparticles (G) and a pyrene-based molecule (py) has been successfully accomplished to give the functionalized nanofillers (G-py). The proposed modification has proven to be a winning solution aimed at safeguarding the graphene’s notable electronic properties, while promoting a more effective nanofiller dispersion attributable to a decrease in viscosity with consequent improvement of the rheological properties of the formulated nanocomposites filled with G-py. The electrical current maps of the G-py based epoxy composites, loaded with filler weight percentages both above and below the electric percolation threshold (EPT), were obtained by tunneling atomic force microscopy (TUNA) technique. The possibility to detect low currents also for the sample at lower concentration (0.1 wt%) confirms the good electrical performance of the nanocomposites and, consequently, the successful performed functionalization. The non-covalent modification significantly improves the thermal stability of the unfunctionalized G of about 70 °C, thus causing an increase in the composite oxidative thermostability since the evolution of CO_2_ shifts to higher values. Moreover, non-covalent functionalization proved to be impactful in imparting an overall enhancement of the nanocomposite mechanical properties due to good bonding between graphene and epoxy matrix, also showing a greater roughness which is decisive in influencing the interface adhesion efficiency.

## 1. Introduction

Graphene is a two-dimensional sp^2^-hybridized carbon nanofiller that has attracted a compelling theoretical and applicative interest in recent years becoming one of the most deeply studied material [1]. Because of its novel properties, such as exceptional thermal conductivity [2], high Young’s modulus [3], and high electrical conductivity [4], graphene is becoming increasingly sought after in fabricating various micro-electrical devices, batteries, supercapacitors, and composites [5,6,7,8,9]. Significant improvements in the final properties can be obtained when graphene is homogeneously dispersed in the matrix and the external load is efficiently transferred through strong filler/polymer interfacial interactions [10]. However, the large surface area of the graphene layers and the strong van der Waals forces inevitably lead to a drastic aggregation of the nanoparticles in the composite matrix. Furthermore, the carbon atoms on the graphene are chemically stable because of the aromatic nature of the bond. As a result, the reinforcing graphene is inert and can interact with the surrounding matrix mainly through van der Waals intermolecular forces, unable to provide an efficient load transfer across the graphene/matrix interface [11]. In this regard, strong interfacial adhesion between graphene-matrix and adequate dispersion of graphene nanoparticles are necessary to meet structural performance for the final multifunctional nanocomposites. Currently, arduous research work has been conducted to modify/functionalize the surface of graphene sheets. The two most common methods adopted toward this purpose involved covalent and non-covalent chemical modifications [12,13]. Covalent functionalization primarily involves classical organic reactions, such as diazonium coupling, cycloaddition [14], substitution, and other reactions such as the enclosure of ionic groups on the surface of graphene. The covalent modifications involve further disruption of the conjugation of the graphene sheets producing hybridized sp^3^ carbon, resulting in the loss of nanofiller electrical conductivity [13,15]. Instead, non-covalent approaches through the use of π–π interactions, van der Waals forces, hydrogen bonding, ionic interactions, or electron–donor-acceptor complexes allow for keeping intact the electrical properties of the graphene material, thus manifesting its driving force in the control and improvement of the properties of graphene and graphene oxide aimed at a real nanotechnological application of two-dimensional (2D) nanofillers [16]. In this work, we explored the effectiveness of functionalization carried out through the π–π stacking interaction between unfunctionalized graphene G and a pyrene derivative, py, to provide the functionalized nanofiller G-py, considering such different aspects as morphology as well as electrical, thermal, dynamic-mechanical, and viscosity properties. The results obtained evidence that the incorporation of non-covalently functionalized G-py in the epoxy resin led to a good dispersion which resulted in a general improvement in structural performance compared to the resin loaded with unfunctionalized G, also ensuring a high electrical conductivity. In particular, with regard to the electrical characterization that we have carried out in this research work, it is worth noting that the direct current (DC) electrical conductivity value recorded for the epoxy nanocomposite loaded with 1 wt% of non-covalently functionalized G-py is 0.1 S/m, while for the same 1 wt% of unfunctionalized G, a value of 4.82 × 10^−3^ S/m is detected. From a direct comparison between these conductivity values, it can be deduced that the non-covalent functionalization of G by pyrene derivative molecules to give G-py, not only preserves the electrical properties of the nanocomposites, but also leads to an increase in the value of the electrical conductivity of two orders of magnitude with respect to the resin loaded with the same 1 wt% of unfunctionalized G. The percentage of 1 wt%, calculated with respect to all the components of the epoxy matrix (R), has been chosen because it is above the electrical percolation threshold, which is in the range 0.025–0.1 wt% for the G based nanocomposites [17]. The unfilled epoxy matrix (R) is characterized by a volume conductivity at room temperature of about 6.00 × 10^−14^ S/m. As main result of the morphological investigation, the chemical functionalization was found to be decisive in preventing the particle agglomeration during the dispersion phase, thus allowing optimization of the preparation process [18,19,20] of the nanofilled samples without, although changing their electrical performance. In this regard, a conductivity mapping at the nanoscale level of the formulated samples loaded with both unfunctionalized G and non-covalently functionalized G-py has been obtained by tunneling atomic force microscopy (TUNA), which allows measuring ultra-low currents ranging from 80 fA to 120 pA [8,21] and also providing information on the achievement or not of the electric percolation threshold (EPT). The formulated materials, being characterized by high thermal stability as well as excellent electrical and mechanical properties, manifest great applicative potentialities in the sector of structural materials.

In addition, the rheological properties of the epoxy samples filled with unfunctionalized G and functionalized G-py nanoparticles are related to the material’s microstructure [20], the dispersion state and shape of nanofillers, and the particle-particle interaction. Together with a deep knowledge of the mechanical and electrical performance of the composites, it is of great importance to understand the rheological behavior for optimizing the manufacturing processes and to obtain information about whether the structure is percolated. Knowledge of rheological properties is beneficial in foretelling the product performance for long-lasting applications, thus giving the chance to refine the processing conditions essential to fabricate superior and trustworthy structural materials [22]. In this regard, the performed functionalization had a significant effect on the rheological behavior of the epoxy nanocomposites as it led to a reduction in viscosity. In fact, the inclusion of a functionalized G-py amount of 0.5 wt% caused the decrease in the complex viscosity of the unfilled epoxy resin R, while instead the same quantity of unfunctionalized G resulted in an increase of its viscosity. In this regard, it is worth noting that the presence of graphene is reported in the literature to usually increase composite suspension viscosity [23,24]. The reduced viscosity due to the functionalization is of crucial importance from the industrial point of view since the use of non-covalently functionalized G-py allows to considerably simplify the steps of the preparation process of the nanocharged epoxy samples, thus favoring also the impregnation of the plies of carbon fiber fabrics. It is well known that nanofilled resins used to impregnate carbon fibers, to be advantageously applied, must be in the nanofiller’s concentration range beyond the electric. The strategy of non-covalent functionalization of the nanofiller can be adopted to solve the non-trivial problem related to the increase of viscosity detected beyond the EPT. The non-covalent functionalization of graphene performed with a pyrene derivative greatly improves the thermal stability of the unfunctionalized G, causing the evolution of carbon dioxide CO_2_ to shift to higher temperature values. Moreover, in this paper, non-covalent functionalization allows obtaining good mechanical properties as a result of an effective bond between the filler and the matrix, thus confirming its ability in acting as a strategic way to control the properties and improve the performance of graphene in various advanced applications [16].

## 2. Experimental

### 2.1. Materials

#### Non-Covalent Functionalization of Graphene Nanoparticles

**Scheme 1:**
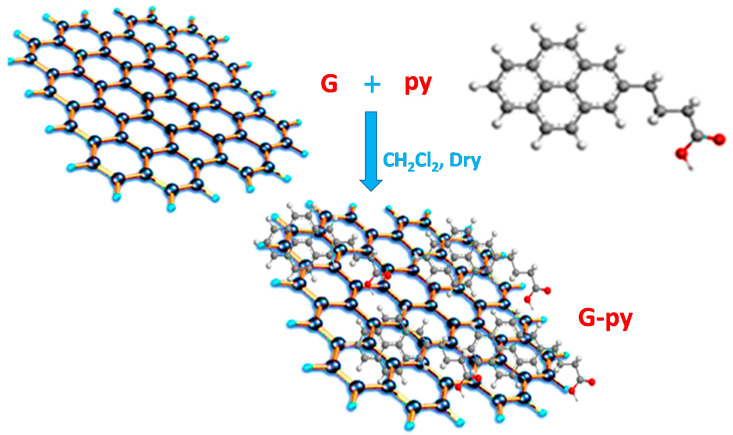
Non-covalent functionalization by stacking.

By exfoliating natural graphite characterized by a high surface area with an average diameter of 500 μm (Asbury graphite grade 3759, Asbury Carbons, New Jersey (NJ)), we obtained conductive graphene nanoparticles (G). In particular, the exfoliation procedure is based on intercalation of natural graphite in a solution of nitric and sulphuric acids, followed by an abrupt treatment at high temperature (900 °C) in a reactor to obtain the expansion of graphene layer spacing [17]. The percentage of the exfoliated phase of nanofiller G is 60%. Its peculiarity consists in having a very high concentration (10 wt%) of carboxylated groups located at the edges of the graphene sheet or graphitic blocks, which are responsible for the formation of self-assembled architectures that lead to the improvement of the mechanical and electrical properties of the final composites [17]. The G nanoparticles in the resin consist of small multilayer stacks of graphene that are from 1 to about 16-nm thick, with diameters ranging from sub-micrometer to few tens of micrometers. The distance between graphitic stacks is about between 5 and 10 nm. G sample contains graphitic blocks composed of a number of layers between 5 and 29 [17]. 1-Pyrenebutyric acid (py) was purchased by Sigma-Aldrich (Milan, MI, Italy). Scheme 1 shows the non-covalent functionalization that was made by mixing in a reaction flask ≈0.100 g of 1-pyrenebutyric acid (py) and ≈1 g of nanocharge G in 50 mL of CH_2_Cl_2_ dry. The functionalized G-py sample (1.080 g) was obtained after filtering, washing with 30 ml of the CH_2_Cl_2_, and drying throughout the night under vacuum the mixture which was kept under stirring for 2 h at room temperature.

### 2.2. Fabrication of Epoxy Samples

By mixing 80 wt% of the tetraglycidylmethylenedianiline (TGMDA) with 20 wt% of the reactive diluent 1-4 butanedioldiglycidyl ether (BDE) which is able to expedite the dispersion step of nanofiller [25], and, then, by adding the hardener 4,4’-diaminodiphenyl sulfone (DDS) in stoichiometric quantity, we have obtained the unfilled epoxy resin identified with the letter R. TGMDA, BDE and DDS were mixed at 120 °C and the unfunctionalized G and functionalized G-py nanoparticles were dispersed by ultrasound for 20 min (Hielscher model UP200S-24KHz high power ultrasonic probe) into the matrix at the following weight percentages of 0.025 wt%, 0.1 wt%, 0.32 wt%, 0.5 wt%, 1 wt%, and 1.8 wt% to manufacture nanocomposites identified with the following code RY%G and RY%G-py, where Y represents the percentage by weight of the unfunctionalized G and functionalized G-py. It is worth noting that the loading concentrations of 0.1 wt% and 1 wt% have been chosen for the electrical characterization by TUNA because they refer to the percentages below and above the electrical percolation threshold (EPT), respectively found for the nanocomposites based on the unfunctionalized G [17]. The epoxy samples were subjected to a two-stage treatment process, namely at 125 °C for 1 h and then at 200 °C for 3 h.

### 2.3. Characterization Methods

A micro-Raman spectrometer Renishaw inVia (Warsash Scientific Pty Ltd, Redfern, Australia) was used to perform a Raman spectral mapping of both the functionalized G-py and the pristine G in the range 100–3200 cm^−1^. The Raman analysis was recorded at room temperature. An excitation wavelength of 514 nm and a laser power of 30 mW was used.

Field emission scanning electron microscope (FESEM) pictures of the two nanofillers G and G-py and their corresponding nanocomposites were acquired using SEM (mod. LEO 1525, Carl Zeiss SMT AG, Oberkochen, Germany). Before the examination by FESEM, thin slices of solid samples were cut and subjected to an etching procedure with an oxidizing solution [8,10,17] to cause the consume of the amorphous resin enclosing the graphene sheets and to display the nanofiller distribution within the polymer matrix more clearly [8,9,17,21,25].

A Keithley 6517A multimeter and an ammeter HP34401A (Loveland, CO, USA) were used to measure the direct current (DC) conductivity values reported in this paper for some of the graphene-based nanocomposites analyzed.

TUNA exploration was accomplished in a contact mode on the graphene-based nanocomposites after the etching procedure. An electrically conductive tip of 20 nm and platinum-coated probes with nominal spring constants of 35 Nm^−1^ were used. TUNA control parameters such as DC sample bias between 1 and 3 V, 1pA/V current sensitivity, 0.500 Hz s^−1^ scan rate, 512 samples/line were set for image acquisition. It should be pointed out that the TUNA nanoelectrical characterization was realised without using conductive silver paste which guarantees suitable electrical contacts of the sample with the ground [8,21]. Bruker software Nanoscope Analysis 1.80 (BuildR1.126200, Digital Instruments, Santa Barbara, CA, USA) was used to perform the graphic processing of the four microscopy images of height, deflection error, friction, and TUNA current, captured concurrently.

A Physica MCR 301 (Anton Paar) rotational rheometer (Anton Paar, GmbH, Ostfildern, Germany) fitted with a parallel plate geometry (50 mm diameter, 1 mm gap) was used to carry out the rheological measurements on the liquid systems, before they undergo the curing process. In particular, in this work, we show the rheological results of the ternary TGMDA/BDE/DDS mixture (labelled R), of the RY%G dispersions, where the Y values are 0.5, 0.75 and 1 wt% of the unfunctionalized graphene content, G, and of the RY%G-py dispersion, where the Y values are 0.5, 0.75 and 1 wt% of G-py.

The linear viscoelastic region was determined by strain sweep tests, at the frequency of 1 rad/s and at the temperatures of 25, 50, and 75 °C.

The linear viscoelastic (LVE) limit for the uncured epoxy matrix R (TBD) is 40% at T = 25 °C, LVE limit = 30% at T = 50 °C, and LVE limit = 10% at T = 75 °C.

The linear viscoelastic limit for R0.5%G based epoxy sample dispersions with unfunctionalized graphene G are: LVE limit for the R0.5%G = 1% at T = 25 °C, LVE limit for the R0.5%G = 2% at T = 50 °C, LVE limit for the R0.5%G = 10% at T = 75 °C.

The linear viscoelastic limit for R0.75%G based epoxy sample dispersions with unfunctionalized graphene G are: LVE limit for the R0.75%G = 0.005% at T = 25 °C, LVE limit for the R0.75%G = 0.07% at T = 50 °C, LVE limit for the R0.75%G = 0.3% at T = 75 °C.

The linear viscoelastic limit for R1%G based epoxy sample dispersions with unfunctionalized graphene G are:

R1%G = 0.002% at T = 25 °C, LVE limit for the R1%G = 0.02% at T = 50 °C, LVE limit for the R1%G = 0.1% at T = 75 °C.

Moreover, the linear viscoelastic limit the R0.5%G-py dispersion with pyrene-functionalized graphene G-py are: LVE limit for the R0.5%G-py = 5% at T = 25 °C, LVE limit for the R0.5%G-py = 5% at T = 50 °C, LVE limit for the R0.5%G-py = 30% at T = 75 °C.

Then, the linear viscoelastic limit the R0.75%G-py dispersion with pyrene-functionalized graphene G-py are: LVE limit for the R0.75%G-py = 5% at T = 25 °C, LVE limit for the R0.75%G-py = 5% at T = 50 °C, LVE limit for the R0.75%G-py = 5% at T = 75 °C.

Finally, the linear viscoelastic limit the R1%G-py dispersion with pyrene-functionalized graphene G-py are: LVE limit for the R1%G-py = 5% at T = 25 °C, LVE limit for the R1%G-py = 5% at T = 50 °C, LVE limit for the R1%G-py = 5% at T = 75 °C.

Frequency sweep tests at 25 °C, 50 °C, and 75 °C were performed, within the linear viscoelastic region, varying the angular frequency from 10^−2^ to 10^2^ rad/s.

Dynamic mechanical properties were measured with a Tritec 2000 DMA (Triton Technology Ltd., Nottinghamshire, UK). The sample dimensions were 2 × 10 × 35 mm^3^. A variable flexural deformation in three points bending mode was applied. The mechanical tests were carried out in a temperature range between −150 °C to 350 °C at the scanning rate of 3 °C min^−2^, frequency of 1 Hz, and displacement amplitude of 0.03 mm.

A Mettler DSC 822 differential scanning calorimeter (Mettler-Toledo, Novate Milanese, Italy) in a flowing nitrogen atmosphere was used to carry out thermal characterization between 0 and 300 °C with a scan rate of 10 °C min^−1^. The Cure Degree (DC) of the samples was assessed using the calorimetric data [21,22,26].

A Mettler TGA/SDTA 851 thermobalance (Mettler-Toledo, Novate Milanese, Italy) was used to carry out the thermogravimetric analysis (TGA). The temperature range between 0 °C and 1000 °C at a 10 °C/min heating rate under both nitrogen and air flows was used to heat the formulated samples.

## 3. Results and Discussion

### 3.1. Raman Characterization of G and G-py Nanofillers

**Figure 1:**
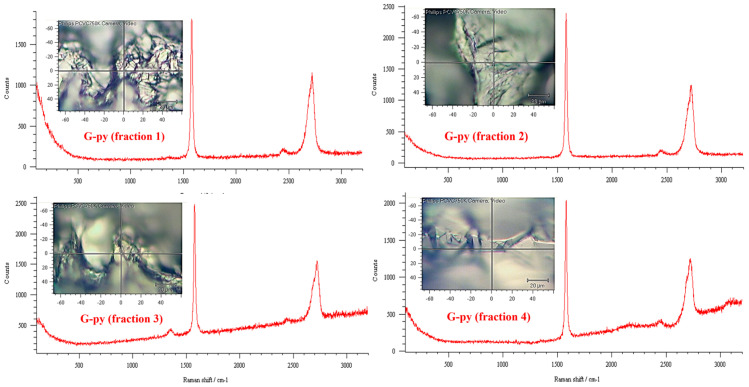
Raman spectral mapping of functionalized G-py nanofiller.

**Figure 2:**
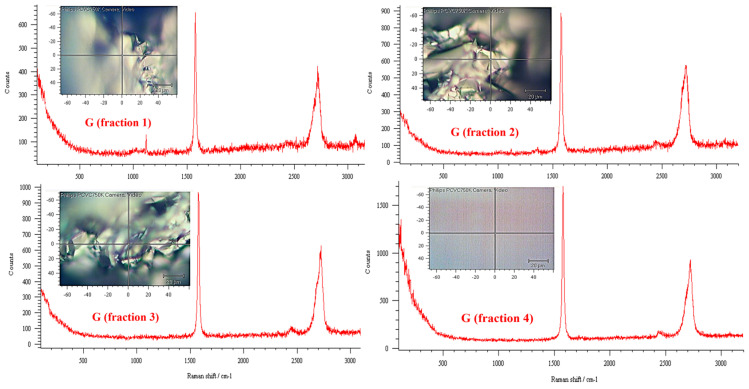
Raman spectral mapping of unfunctionalized G nanofiller.

The functionalized G-py nanoparticles were characterized by Raman spectroscopy. The G-py spectra were compared with those of unfunctionalized G to obtain information on the degree of structural ordering and therefore the bonding states of carbons in the graphene structure. Figure 1 and Figure 2 show the Raman spectra of functionalized G-py and unfunctionalized G nanoparticles, respectively. In particular, to ensure the reproducibility of the Raman measurements, the spectra have been collected on distinct fractions of G and G-py. More precisely, for this purpose, the Raman spectral mapping on different fractions of the two samples was performed. For simplicity, only four spectra corresponding to four fractions of each type of nanofiller are shown here (see Figure 1 and Figure 2).

In the Raman spectrum first-order region (1100–1800 wavenumbers), we can observe the main graphite band, named the G band, at ~1580 cm^−1^, distinguishable with different intensity for all the samples. In the case of less crystalline graphite, further bands at ~1350–1355 cm^−1^, 1500 cm^−1^, and 1622 cm^−1^ are detectable. The D band at ~1350 cm^−1^ is induced by the disordered structure of graphene sheets. The presence in the second-order region (2200–3300 wavenumbers) of the 2D or G’ band at ~2700 cm^−1^, due to overtones and combinations of the disorder-induced bands, is characteristic of graphite crystallinities. G-py sample exhibits a pattern similar to the G sample. For both nanofillers, the very small difference in the intensity of the bands relative to each sample is due to the local region where the spectrum was detected. This difference is due to the fact that on the edge of the nanoparticles a greater number of defects is present. A general conclusion of this investigation is that the non-covalent modification did not destroy the layered structure of pristine G nanofiller, thus proving that non-covalently functionalized G-py can be successfully employed to formulate the functional nanocomposites for structural applications.

### 3.2. FESEM Characterization of G and G-py Nanofillers and Their Corresponding Nanocomposites

**Figure 3:**
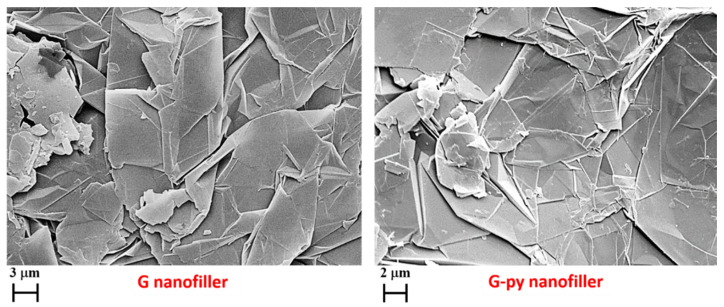
FESEM images of unfunctionalized G and functionalized G-py nanofillers.

**Figure 4:**
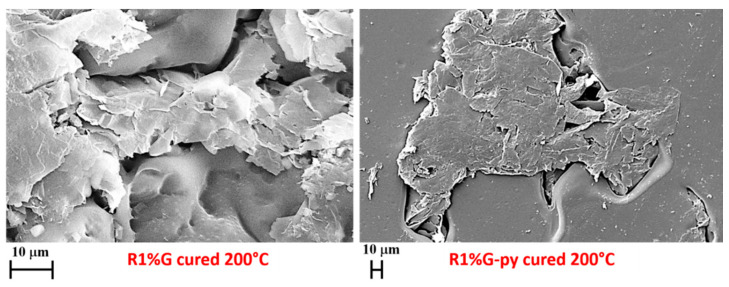
FESEM pictures of the samples R1%G and R1%G-py cured up to 200 °C.

FESEM pictures of the pristine G and functionalized G-py nanofillers are shown in Figure 3 and FESEM pictures of the fracture surfaces of the cured R1%G and R1%G-py samples are shown in Figure 4.

We can clearly observe that for both G and G-py samples, the exfoliated graphite sheets exhibit a highly fluffy morphology representative of thermally treated graphite. Furthermore, FESEM images show the folds and wrinkles present on the surface of the relatively large (about several micrometers) and uniform graphene platelets. This confirms, as the Raman analysis, that the graphene-layered structure is not destroyed by non-covalent functionalization.

The dispersion of the nanofiller in the polymeric matrix was analysed by the FESEM technique on sample surfaces that have previously undergone the acid attack of an etching solution which, by consuming the resin, allowed to discover the nanocharge. From Figure 4, we can clearly detect the uniform dispersion of both unfunctionalized G and functionalized G-py nanoparticles inside the host resin, where only a few graphene sheets are distinguishable on the surface, coming out of worn resin layers. The etching procedure was found to be truly effective because it allowed both to evaluate the dispersion uniformity of the graphene nanoparticles and to detect their strong interconnections with the polymer matrix thus demonstrating the great ability of the nanoparticles G, and G-py to increase the filler-matrix interactions by resisting well the acid attack of the oxidizing solution. The effect of the resistant anchorage to the epoxy matrix which, preventing the portion of resin around the nanofiller from being consumed, allows a more efficacious charge transfer which results in excellent mechanical performance of the analyzed nanocomposites, appears particularly noticeable for the functionalized nanoparticles G-py.

### 3.3. TUNA Characterization of Graphene-Based Nanocomposites

**Figure 5:**
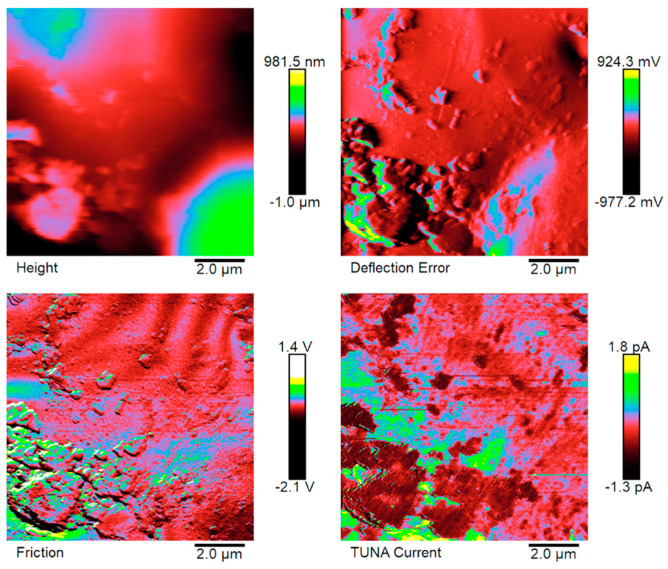
TUNA pictures of the R0.1%G-py fracture surface.

**Figure 6:**
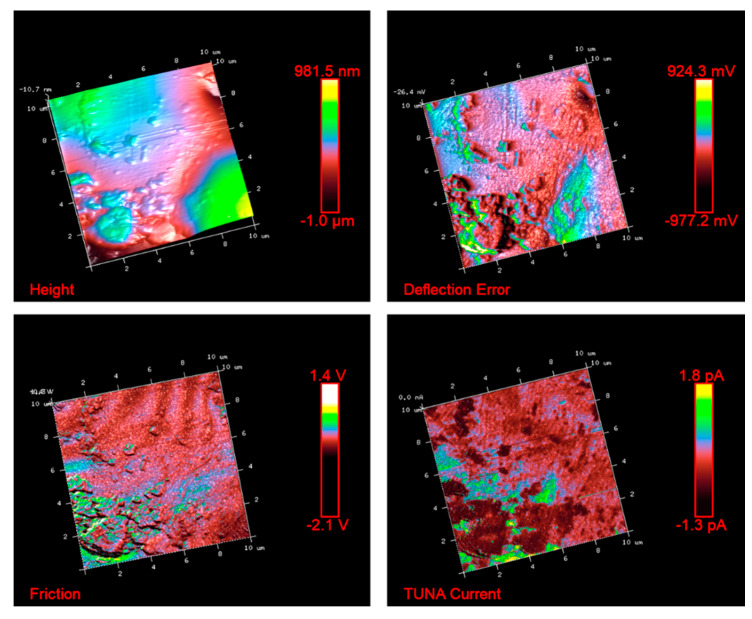
TUNA pictures of the 3D shapes (corresponding to the images reported in Figure 5) of the R0.1%G-py fracture surface.

**Figure 7:**
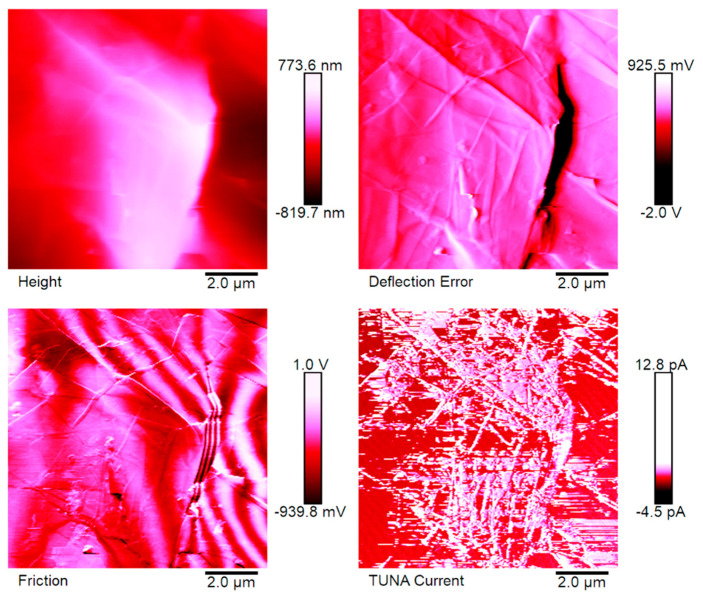
TUNA pictures of the R1%G-py fracture surface.

**Figure 8:**
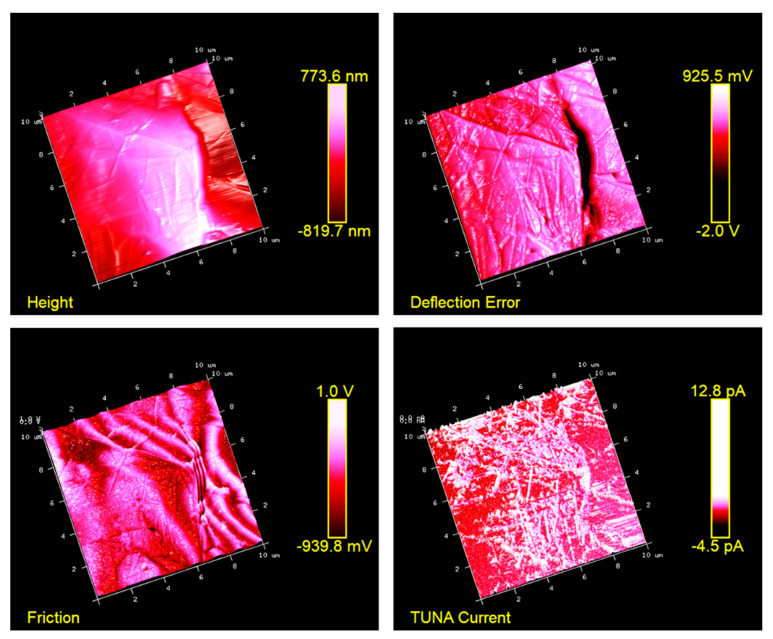
TUNA pictures of the 3D shapes (corresponding to the images reported in Figure 7) of the R1%G-py fracture surface.

Figure 5, Figure 6, Figure 7 and Figure 8 show the TUNA pictures and the analogous three-dimensional (3D) shapes of the fracture surfaces of the two etched epoxy samples loaded with 0.1 wt% and 1 wt% of functionalized nanofiller G-py, where it is possible to clearly observe the carbon nanostructure distribution. Four kinds of TUNA pictures for each sample analyzed are displayed. Precisely, they are the following: Height (or topography), Deflection Error, Friction, and TUNA Current images, which show the morphological features of the nanocomposite at different loadings of functionalized nanofiller and furnish correlative information that effectively support the reader in understanding the observed electrical performance. The TUNA images clearly provide an overview on the effect produced by the non-covalent functionalization of the graphene nanosheets. This kind of functionalization is able to improve the interaction capability of the nanoparticles with the hosting matrix at level of the interfacial nanodomains, where they are firmly connected, originating an extensive electrically conductive network (see sample 1 wt% of G-py). This effect is strongly evident for the nanocomposite loaded with the higher percentage of functionalized filler. Morphological details related to the nature of the nanofiller are observable in Figure 7 and Figure 8. The nanofiller surface appears similar to the corrugated texture of a drapery (see deflection error images of Figure 7 and Figure 8). It is clearly visible the accentuated contrast in the brilliance of the colors, clearly detectable in the TUNA current images of Figure 7 and Figure 8, as evident on the sidebar, which associates the colors to the different recorded current values, providing an effective mapping of the nanodomains with areas of high current density, where the graphene nanolayers seem to be almost “fused” with the epoxy resin. In any case, the possibility of detecting measurable currents over the entire investigated area of the sample highlights high electrical conductivity values of the G-py-based nanocomposite and the success of the functionalization carried out by choosing the py compound. Consideration of the TUNA current image of the two nanocomposites allows for confirmation of the presence of the conductive nanoparticle, but for the sample at the lower concentration of the nanofiller, the electrical conductivity is reduced with respect to the sample at the higher concentration of the nanofiller. In particular, for the lower concentration, in the majority of the sample domains, no electrically conductive paths are observed. In fact, in many domains, the nanoparticles seem to be characterized by poor interconnections. For the samples R0.1%G-py and R1%G-py, currents ranging from 1.3 pA to 1.8 pA (see TUNA current images in Figure 5 and Figure 6) and 4.5 pA to 12.8 pA (see TUNA current images in Figure 7 and Figure 8) were detected, respectively. These values clearly indicate that the electrical percolation threshold (EPT) has not yet been reached for the sample at the lower concentration. However, the possibility to detect low currents, also for the sample at a lower concentration, confirms the good electrical performance shown by the non-covalently functionalized G-py epoxy system. It is noteworthy that for the R1%G-py sample, for which a value of 0.1 S/m is detected, intense conductive paths are easily detectable in the TUNA current images, as deducible by the intense color contrast observable in Figure 7 and Figure 8. Furthermore, the non-covalent functionalization has led to an increase of two orders of magnitude with respect to the electrical conductivity value of 4.82 × 10^−3^ S/m obtained for the G based epoxy nanocomposite R1%G, loaded at the same loading concentration of 1 wt%, which corresponds to the percentage above the electrical percolation threshold. The enhancement in the electrical conductivity is most likely due to a better dispersion of the filler and the nature of the functionalizing py compound.

Surface texture knowledge is an important question when aiming to comprehend the nature of the material surface and it is of primary importance when there is interest in verifying the adhesion effectiveness at the interface between the matrix and the nanoparticles. The mechanical interaction associated with the surface roughness can be effectively evaluated by means of TUNA technique which allows deriving quantitative measurements of roughness using two of the most significant height parameters, namely the roughness average (Ra), which is the arithmetic mean of the absolute values of the height of the surface profile, and the root mean square roughness (Rq) which is analogous to the roughness average (Ra), with the only difference being the mean squared absolute values of surface roughness profile. The Rq is more sensitive to peaks and valleys than the average roughness due to the squaring of the amplitude in its calculation [27].

**Figure 9:**
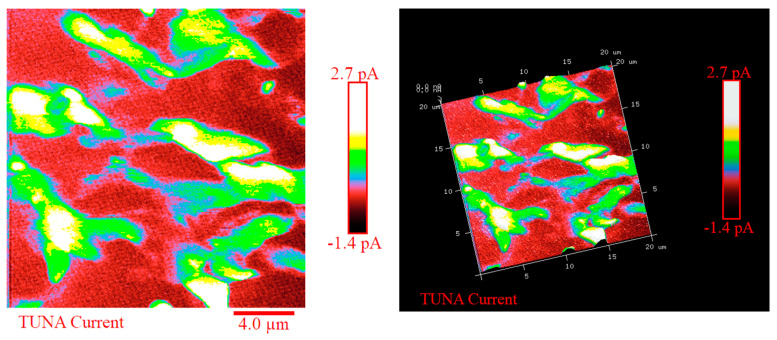
TUNA Current pictures (from left to right: 2D and 3D shapes) of the R1.8%G fracture surface.

In this work, nanoscale roughness Ra and Rq values of the prepared composites R0.1%G-py (see Figure 5 and Figure 6) and R1%G-py (see Figure 7 and Figure 8) have been calculated from the TUNA images using Bruker software Nanoscope Analysis 1.80 (BuildR1.126200). For comparison, the Ra and Rq values, calculated for the TUNA Current micrographs (2D profile on the left and 3D profile on the right), of the fracture surface of the R1.8%G sample (see Figure 9) are also reported. These pictures unequivocally show the presence of graphene sheets uniformly dispersed in the polymer matrix, thus ensuring effective conductive paths with high current values ranging from 1.4 pA to 2.7 Pa. It is worth noting that the nanocomposite R1.8%G, being beyond the EPT, is characterized by an electrical conductivity value of 0.096 S m^−1^. Regarding the nanocomposite R0.1%G-py, the roughness values assessed for the TUNA pictures shown in the Figure 5 and Figure 6 are as follows: Ra 183 nm and Rq 243 nm from Height profile, Ra 0.127 V and Rq 0.206 V from Deflection profile, Ra 0.176 V and Rq 0.539 V from Friction profile, Ra 0.298 pA and Rq 0.405 pA from TUNA current profile.

**Figure 10:**
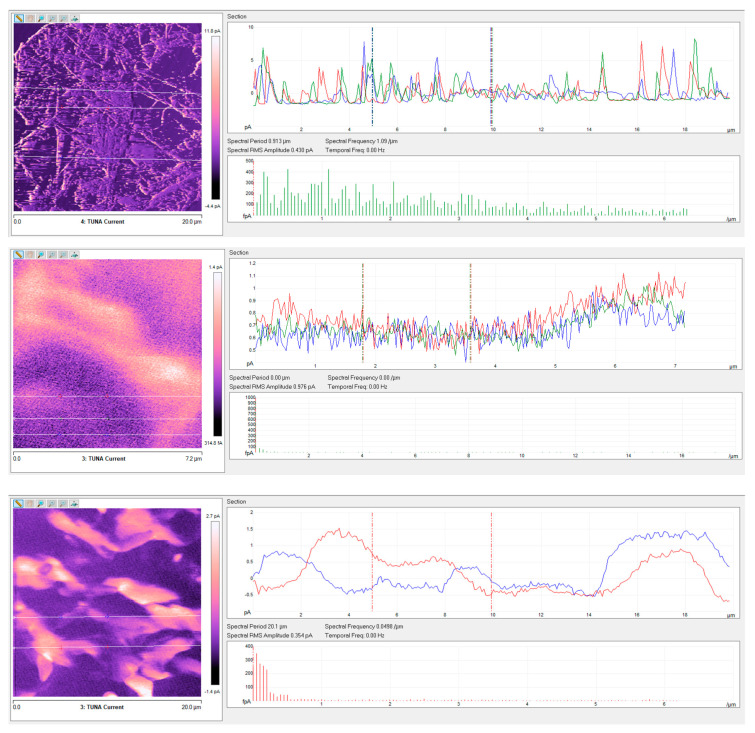
TUNA Current pictures and current variations of the fracture surface of the R1%G-py (see top image), R1%G (see middle image), and R1.8%G (see bottom image) samples.

Regarding the nanocomposite R1%G-py, the roughness values assessed for the TUNA pictures shown in the Figure 7 and Figure 8 are as follows: Ra 222 nm and Rq 269 nm from height profile, Ra 0.153 V and Rq 0.277 V from deflection profile, Ra 0.218 V and Rq 0.667 V from friction profile, Ra 1.39 pA and Rq 2.11 pA from TUNA current profile. The nanocomposite R1.8%G show Ra 0.518 pA and Rq 0.642 pA from TUNA current profile. From these roughness values, we can deduce that the roughness values Ra and Rq detected for the nanocomposites based on non-covalently functionalized G-py undergo an increase in passing from 0.1wt% of filler (below EPT) to 1 wt% of filler, as expected. In fact, increasing the amount of nanofiller, the fracture surface becomes increasingly bumpy because of the graphitic layers and therefore of the heterogeneity of the sample during the fracture. Analyzing the roughness measurements, it is possible to investigate the dispersion state of the nanofiller in the matrix. At this aim, the top image in Figure 10, corresponding to the TUNA current picture of the sample loaded with 1.0 wt% of functionalized filler G-py (sample R1%G-py), is exemplary to illustrate as the dispersion state of the filler can be deduced from this kind of investigation. In particular, on the TUNA current image on the left side, it is possible to analyze the change in the current along the three linear profiles on the white lines on the image. It is possible to observe the changes in the TUNA current along the three lines on the left side (see green, red and blue graphics on the right). The frequency of the changes due to filler/matrix alternations along the three lines is fairly regular. This is evidence of the good distribution reached. The middle and bottom images in Figure 10 show the change in the current along the linear profiles on the white lines on the TUNA current pictures of the samples containing the unfunctionalized filler G, that is samples R1.0%G and R1.8%G, respectively. It is worth noting that, in the case of the sample R1.0%G (see middle image in Figure 10), the current variations are smaller because the sample, as mentioned before, is less conductive. Nonetheless, major relative unevenness can be seen. The greatest inhomogeneity in the distribution of graphite blocks is recorded for the R1.8%G (see bottom image in Figure 10), which is due to the highest loading of the unfunctionalized filler G. It is also possible to observe that in this last case, the graphitic layers tend to reassemble in blocks of higher dimension.

### 3.4. Dynamic Mechanical Analysis (DMA) of Graphene-Based Nanocomposites

In order to fully understand the influence of the non-covalently functionalized carbon nanofiller G-py on the mechanical behavior of the resin R and G-based nanocomposites, DMA was performed. In a context in which the hydrophobic nature of graphene makes it incompatible with most organic polymers and tend to irreversibly agglomerate itself, the modification of graphene nanoparticles by non-covalent method is becoming increasingly significant for the solution of this problem. In this regard, particular importance is given to π−π interactions because of their strength, comparable to covalent bonding [28] but with the benefit that excellent electrical conductivity for the conjugated structure of graphene can be preserved in this way. Moreover, since G derivatives usually have excellent thermal, electrical, and mechanical, properties, it is possible to exploit these properties by incorporating them in polymer composites. For the ideal incorporation of graphenic nanostructures into polymer matrices, non-covalent interactions, which determine the homogeneity of the composite and the extent of the cooperation between the two components, play a crucial role in preventing the formation of agglomerates and therefore ensuring good mechanical performance of the final composite through an effective dispersion of the nanofiller. In this regard, an amazing example is given by the establishment of π−π interactions between graphenic derivatives and polymers that contain aromatic rings. A repeating aromatic polymer unit can strongly bind graphenic monolayers leading to highly homogeneous polymer composites with enhanced electrical, thermal, and mechanical properties [16,29,30,31,32].

**Figure 11:**
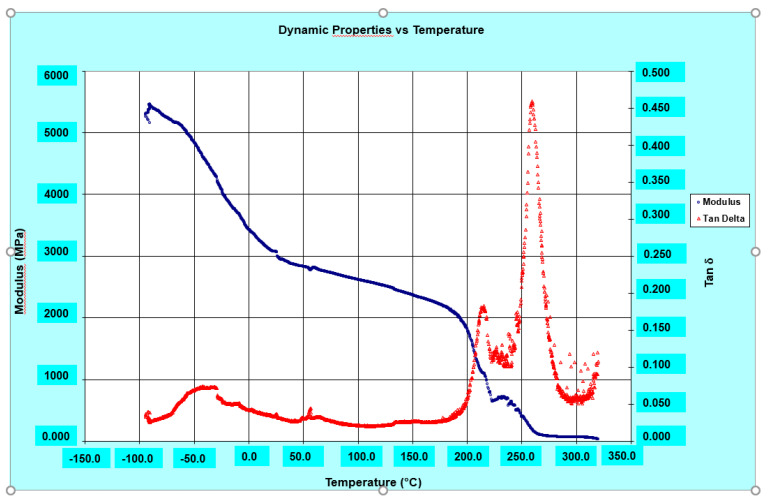
Loss factor (tanδ) and Storage modulus of the R0.5%G-py nanocomposite.

**Figure 12:**
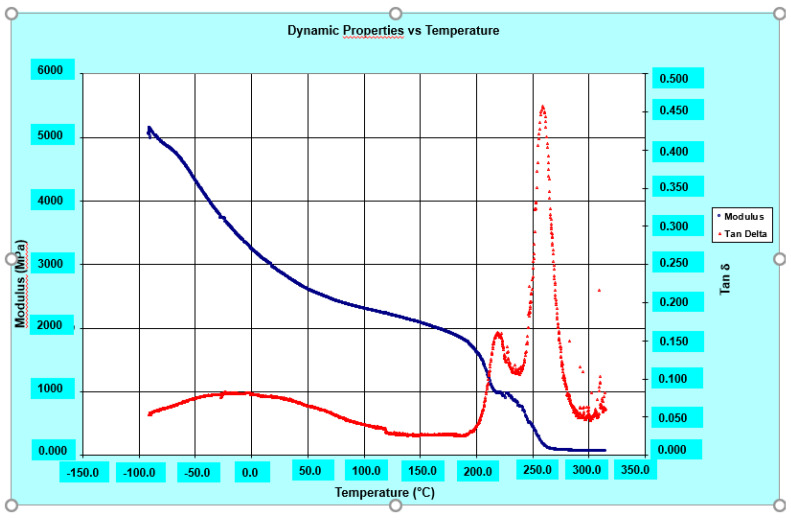
Loss factor (tanδ) and Storage modulus of the R0.5%G nanocomposite.

**Figure 13:**
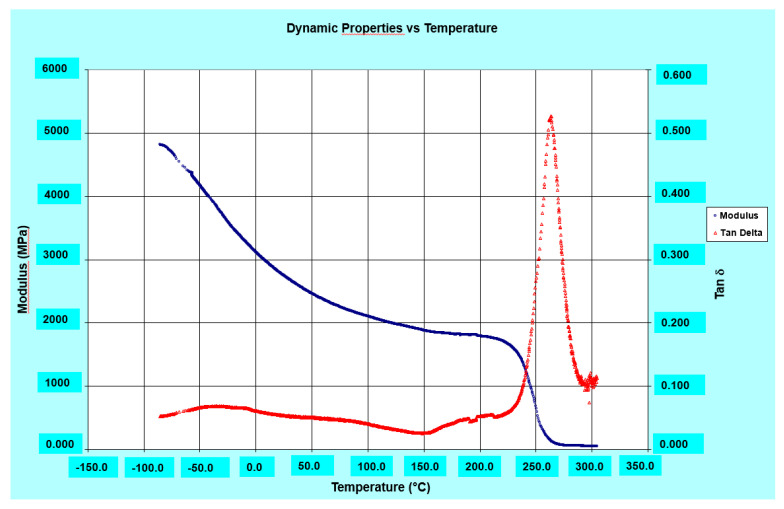
Loss factor (tanδ) and Storage modulus of the unfilled epoxy matrix R.

The DMA results show good values both of storage modulus, even higher than 2000 MPa up to 220 °C, and of glass transition temperature (T_g_) which is around 264 °C for the R0.5%G-py nanocomposite (see Figure 11), 266 °C for the R0.5%G nanocomposite (see Figure 12), and 262 °C for the resin R (see Figure 13). It can be observed that both the unfunctionalized G and functionalized G-py produce a second phase with a lower T_g_ characterized by increased mobility of the portions of polymer chains which are in closer contact with the nanofillers. It is important to highlight how, for the graphene-based nanoparticles that we have used in this work, the exfoliation degree and edge carboxylated groups play a key role in giving rise to the self-assembled architectures capable of promoting the EPT paths and the attractive/covalent interactions with the epoxy matrix [9].

### 3.5. Differential Scanning Calorimetry (DSC) Investigation and Thermogravimetric Analysis (TGA) of G and G-py Nanofillers and Their Corresponding Nanocomposites

**Figure 14:**
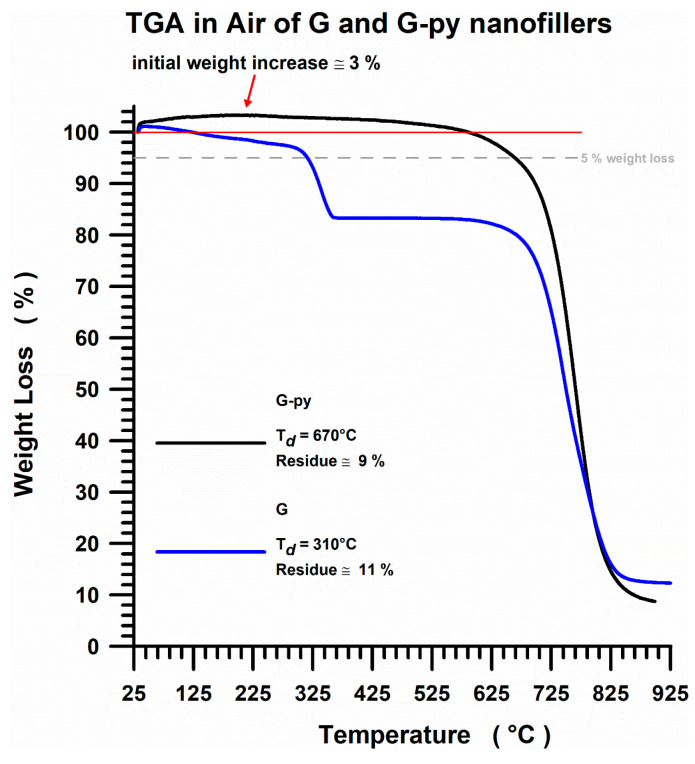
TGA curves in air of G and G-py nanofillers.

In order to better understand and interpret the thermogravimetric data for the different analyzed systems, the TGA trend (with the thermodegradation temperatures T_d_ and residue values) in air of the unfunctionalized G and non-covalently functionalized G-py nanofillers is shown in Figure 14.

From the TGA curves, it can be clearly seen that for the functionalized sample G-py (see the black curve) the beginning of the thermodegradation phenomenon moves to a higher temperature of about 70 °C compared to unfunctionalized G (see the blue curve). More precisely, the non-covalent functionalization of graphene with pyrene derivative significantly improves the thermal stability of the starting unfunctionalized nanocharge G, effectively increasing the oxidative thermostability of the composite because the evolution of CO_2_ shifts to higher values. The thermodegradation temperatures T_d_ are 310 °C for G sample and 670 °C for G-py sample, while instead, a residue of 9% and a residue of 11% are recorded for samples G and G-py, respectively. The functionalized filler G-py also shows an initial weight increase of about 3%. Repeated measurements showed a very slight increase in weight (2–3%). This increase is most likely due to strong adsorption of nitrogen molecules, which are geometrically linear and characterized by small dimensions, on and among thin functionalized graphene layers.

**Figure 15:**
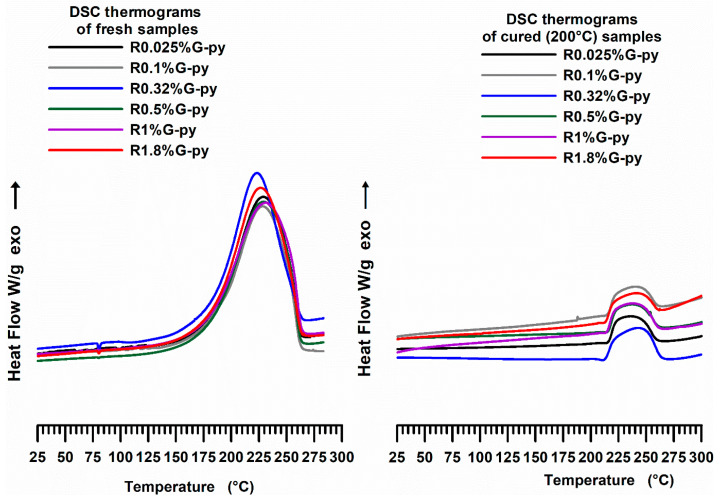
DSC curves of the epoxy formulations (fresh—first run and cured at 200°C—second run) containing different G-py weight percentages.

The curing degree (DC) has been gained in the dynamic and isothermal regime. Figure 15 shows DSC thermograms of the G-py based epoxy formulations: fresh—first run (dynamic regime) and cured at 200 °C—second run (isothermal regime).

**Figure 16:**
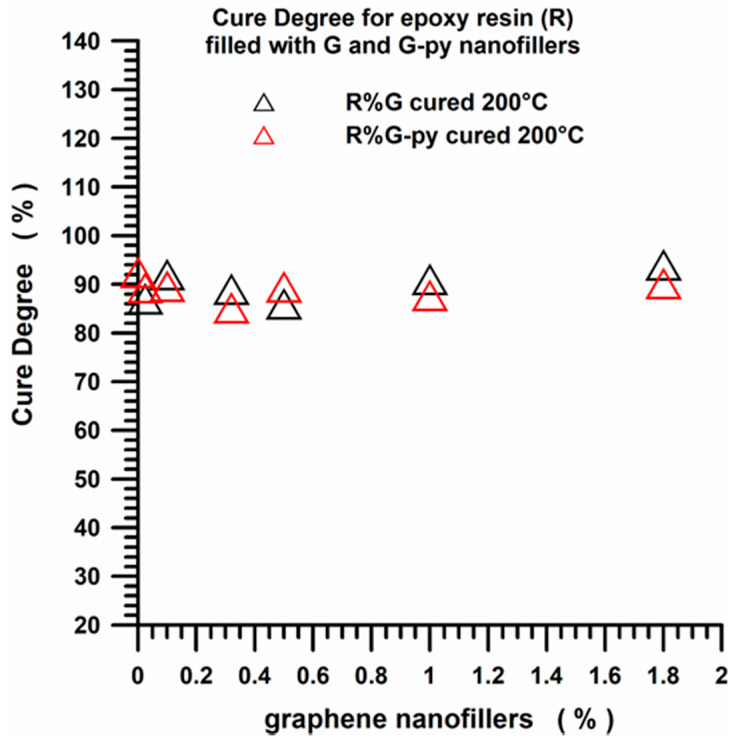
DC values of the pristine G and functionalized G-py based nanocomposites cured under isothermal heating conditions.

For all the analyzed formulations, the curing reactions are active in the range between 125 °C and 250 °C in the dynamic regime. DC values of the pristine G and functionalized G-py based nanocomposites cured under isothermal heating conditions, shown in Figure 16, indicate that all the formulations have a mean value of the curing degree of about 90%, which fully complies with the requirements imposed by the aviation industry.

**Figure 17:**
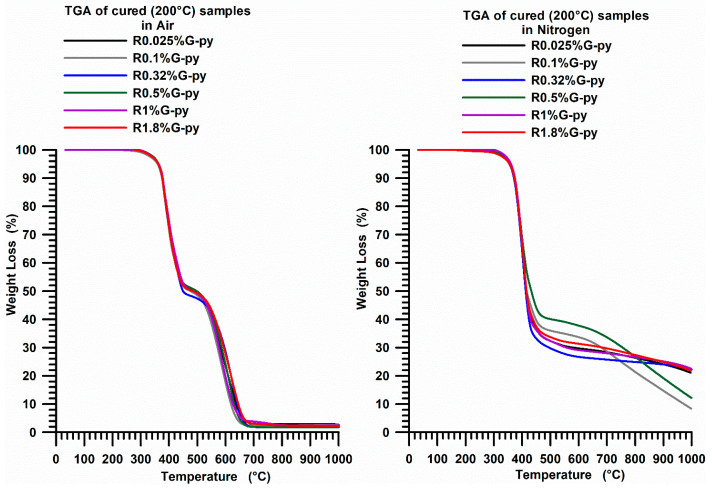
TGA curves in air and in nitrogen of the G-py based nanocomposites cured up to 200 °C.

Figure 17 shows the TGA curves of the cured epoxy formulations filled with different percentages by weight of G-py nanofiller. The samples (after the curing cycle) show two weight loss steps (both in air and in nitrogen) at temperatures of 330 °C and 490 °C while the final residue (at 670 °C) is about 3% in air and about 23% in nitrogen.

**Figure 18:**
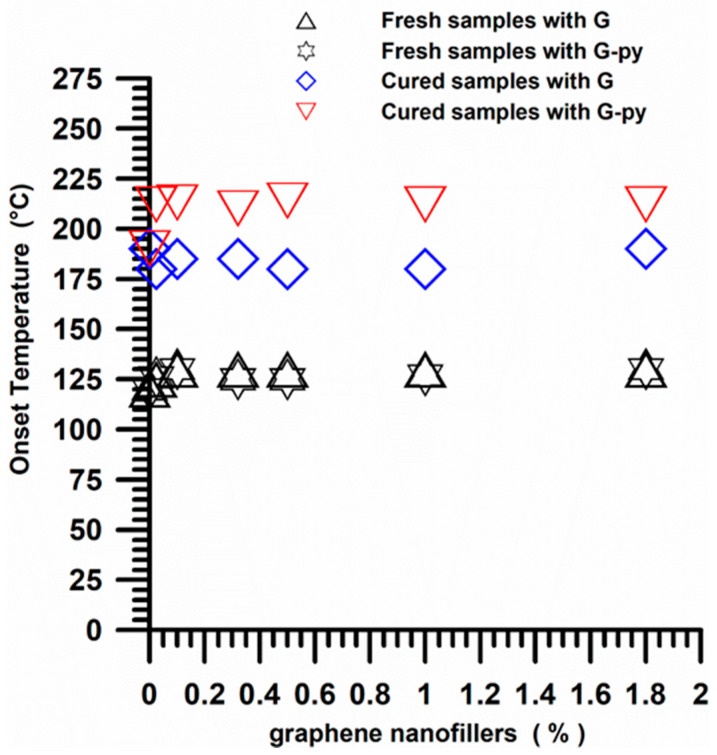
Cross-link onset temperature trend for the fresh (uncured) and cured epoxy nanocomposites with different weight percentages of G and G-py nanoparticles.

Figure 18 shows the cross-link onset temperature trend for the fresh (uncured) and cured epoxy samples filled with pristine G and functionalized G-py nanoparticles. We can observe that for all the fresh samples at different weight percentages of the two nanofillers, the beginning of cross-linking (onset temperature) occurs at about 125 °C. On the contrary, samples cured at 200 °C, undergoing only a “residual cure” process, show onsets which are triggered at about 180 °C for the samples containing both the pristine and the functionalized G.

**Figure 19:**
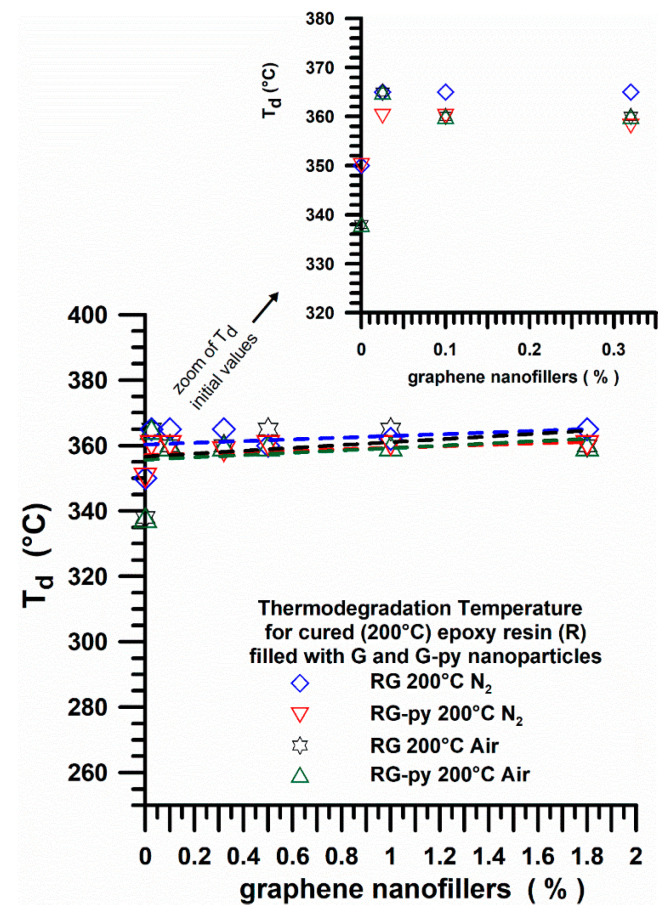
Thermodegradation temperature (in air and nitrogen) for cured (200 °C) epoxy resin (R) filled with G and G-py nanoparticles at different percentages by weight.

Figure 19 shows the thermodegradation temperature (T_d_) (in air and nitrogen) for cured (200 °C) epoxy resin (R) filled with G and G-py nanoparticles at different percentages by weight. In particular, the thermodegradation temperature (T_d_) referred to a sample weight loss of 5% for all the samples containing both pristine G and functionalized G-py nanofillers.

Samples cured in the oven at 200 °C show similar values of T_d_ at about 360 °C in both inert and oxidative environment, as it can be clearly seen by observing the graph relating to the zoom of the initial values of T_d_.

### 3.6. Rheological Analysis of Graphene-Based Nanocomposites

**Figure 20:**
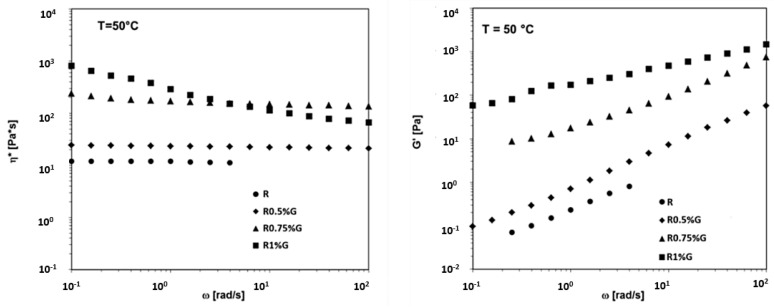
Rheological properties at T = 50 °C for the R uncured epoxy matrix, R0.5%G, R0.75%G and the R1%G by wt liquid dispersions.

The rheological results for the liquid dispersions containing 0.5 wt%, 0.75 wt% and 1 wt% of the unfunctionalized (G) graphene nanoparticles in the R uncured resin, at the temperature of 50 °C, are shown in Figure 20. In particular, Figure 20 shows the complex viscosity (η*) and the storage modulus (G’) vs frequency (ω) at T = 50 °C for the R uncured epoxy matrix, the R0.5%G, R0.75%G, and the R1%G by wt liquid dispersions. We can observe that, when unfunctionalized graphene G with carboxylated groups at the nanoparticle edges is used in the epoxy matrix, at the temperature of 50 °C, the unfilled resin and the mixtures containing up to 0.75 wt% of nanofiller manifest a Newtonian behavior. A shear thinning behavior occurs at the nanofiller content of 1 wt%, indicating a percolated structure.

**Figure 21:**
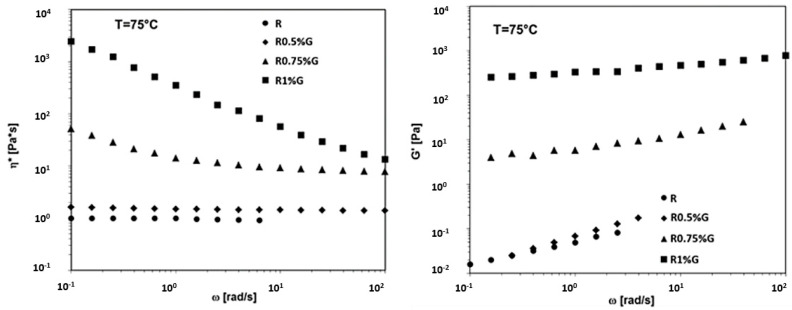
Rheological properties at T = 75 °C for the R uncured epoxy matrix, the R0.5%G, R0.75%G and the R1%G by wt liquid dispersions.

In Figure 21, the complex viscosity (η*) and the storage modulus (G’) vs frequency (ω) at T=75°C for the R uncured epoxy matrix, the R0.5%G, R0.75%G, and the R1%G by wt liquid dispersions are reported. From the graphs shown in Figure 21, it can be clearly seen that, increasing the temperature to 75 °C, the shear thinning behavior in the complex viscosity and a clear plateau in G’ are observed in the epoxy/G liquid dispersions for the lower content of 0.75 wt% of G.

**Figure 22:**
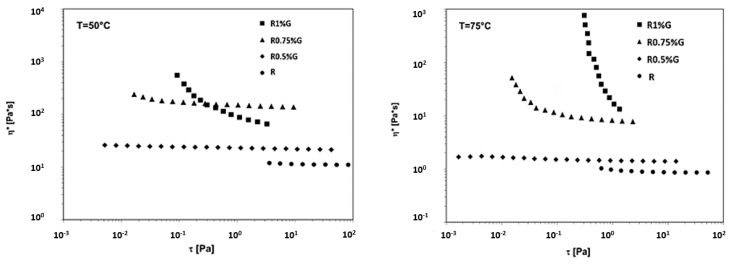
Complex viscosity vs shear stress at T = 50 °C and T = 75 °C for the R uncured epoxy matrix, the R0.5%G, R0.75%G and the R1%G by wt liquid dispersions.

The rheological results can also be analysed in terms of complex viscosity (η*) vs shear stress (τ) and they are shown in Figure 22 for the R uncured epoxy matrix and for the liquid dispersions containing 0.5 wt%, 0.75 wt%, and 1wt% of the unfunctionalized (G) graphene nanoparticles in the R uncured matrix, at the temperatures of 50 °C and 75 °C.

From the graphs shown in Figure 22 we can deduce that, in the case of the epoxy/G dispersions, the yield stress occurs for the content of 1 wt% G at T = 50 °C, while it is observed for the lower content of 0.75 wt% G increasing the temperature to 75 °C. The rheological results reported in Figure 20, Figure 21 and Figure 22, then, clearly show that a lower percolation threshold, indicating a stronger graphene network, is observed as the temperature rises.

Moreover, it is noteworthy that the presence of edge-carboxylation of the graphitic material decreases the rheological percolation threshold to values similar to those observed for the mono-dimensional MWCNTs fillers [33].

**Figure 23:**
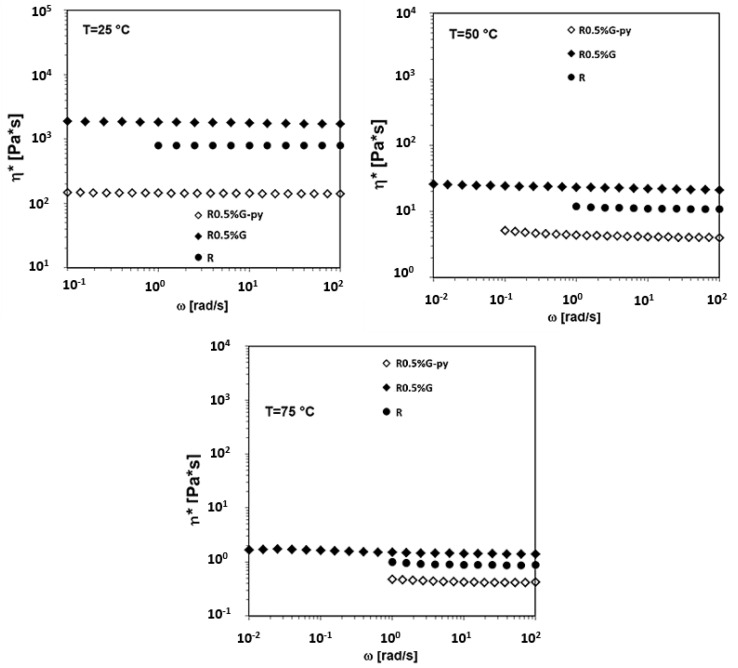
Complex viscosity vs frequency at T = 25 °C, T = 50 °C and T = 75 °C for the R uncured epoxy matrix, the R0.5%G and the R0.5%G-py by wt liquid dispersions.

Finally, the complex viscosity (η*) vs. frequency (ω) for the liquid dispersions containing an amount of 0.5 wt% of the unfunctionalized (G) and functionalized (G-py) graphene nanoparticles in the R uncured matrix, at the temperatures of 25 °C, 50 °C, and 75 °C, are compared in Figure 23. In all cases, it is observed that the inclusion of unfunctionalized G in the epoxy uncured matrix R determines an increase in the viscosity of the R sample, while the inclusion of the functionalized (G-py) graphene nanoparticles in the R uncured matrix determines a significant decrease of the complex viscosity in the whole frequency range investigated. This result represents an important issue in the industrial process of preparation of epoxy nanocomposites.

## 4. Conclusions

In this work, we have demonstrated that the non-covalent functionalization including mainly π–π interactions and hydrogen bonding represents a tactical way to control the properties and ameliorate the fulfillment of graphene nanoparticles characterized by carboxylated groups at the edge of graphene layers or graphitic blocks in advanced applications. Understanding the rheological behavior is of great importance for optimizing the manufacturing process of the structural nanofilled epoxy resins, where the amount of graphene is over the electrical percolation threshold (EPT) and for obtaining information about whether the structure is percolated. From the direct comparison between the direct current electrical conductivity values, we have found that the non-covalent functionalization of graphene G by pyrene derivative molecule, giving G-py sample, not only preserves the electrical properties of the nanocomposites, but also leads to an increase in the electrical conductivity value of two orders of magnitude with respect to the resin loaded with the same percentage of 1% by wt of unfunctionalized G nanofiller. The enhancement in the electrical conductivity is most likely due both to a more effective dispersion of the nanofiller in the matrix and the nature of the py functionalizing compound. In fact, the py compound is characterized by a structural nature very similar to a small portion of graphene. This small part of py compound may locally compensate for the reduced attitude in the electron conduction of zones of nanofiller characterized by structural defects (with a higher percentage of carbon atoms hybridized sp^3^). TUNA analysis was carried out using G-py weight percentages both below and above the EPT in order to investigate the electrical behavior of the conductive nanodomains of the epoxy/graphene systems. In particular, for the lowest concentration equal to 0.1 wt% of G-py, in most of the sample domains, no electrically conductive paths are observed, while for the highest concentration equal to 1 wt% of G -py, the presence of a conductive network at the nanoscale level with efficient adhesion to the interface clearly indicates that it is above the EPT. It is important to emphasize that the possibility to detect low currents also for the sample at lower concentration (0.1 wt%) confirms the good electrical performance of the nanocomposites and, consequently, the successful performed functionalization. These excellent electrical results are in perfect agreement with the rheological results. In fact, the inclusion of a functionalized G-py nanofiller amount of 0.5 wt% caused the decrease in the complex viscosity of the unfilled epoxy resin R, while instead the same quantity of unfunctionalized nanofiller G resulted in an increase of its viscosity. This aspect is of crucial importance from the industrial point of view since the use of non-covalently functionalized G-py allows to considerably simplify the steps of the preparation process of the nanocharged epoxy samples, thus favoring also the impregnation of the plies of carbon fiber fabrics. This is a problem that is by no means insignificant if we consider that nanofilled aeronautical resins used to impregnate carbon fibers contain a percentage of nano-charges capable of producing samples in the nanofiller’s concentration range beyond the EPT. A significant improvement in the thermal stability of the unfunctionalized graphene G of about 70 °C was registered, thus determining an increase in the composite oxidative thermostability since the evolution of CO_2_ shifts to higher values. Moreover, non-covalent functionalization proved to be particularly effective in conferring outstanding mechanical properties on the nanocomposites thanks to the strong interfacial adhesion between graphene-matrix and satisfactory dispersion of graphene nanoparticles inside the epoxy matrix.
